# Implementation of a universal rotavirus vaccination program: comparison of two delivery systems

**DOI:** 10.1186/1471-2458-14-908

**Published:** 2014-09-02

**Authors:** Mitchell Zelman, Carolyn Sanford, Anne Neatby, Beth A Halperin, Donna MacDougall, Corinne Rowswell, Joanne M Langley, Scott A Halperin, Maritime Universal Rotavirus Vaccination Program (MURVP)

**Affiliations:** Department of Health and Wellness, Charlottetown, Prince Edward Island Canada; Canadian Center for Vaccinology, Halifax, Nova Scotia Canada; Department of Pediatrics, Dalhousie University, Halifax, Nova Scotia Canada; IWK Health Centre, Halifax, Nova Scotia Canada; School of Nursing, Saint Francis Xavier University, Antigonish, Nova Scotia Canada

**Keywords:** Rotavirus infection, Rotavirus vaccine, Immunization program, Immunization delivery models, Program evaluation

## Abstract

**Background:**

Rotavirus vaccine is recommended for all infants in Canada. To evaluate the logistics of implementing a universal rotavirus vaccination program, we compared the effectiveness of program implementation in jurisdictions with either a physician-administered or public health nurse-administered program.

**Methods:**

All infants born between October 1, 2010 and September 30, 2012 in Prince Edward Island and Nova Scotia’s Capital District Health Authority were eligible for the vaccination program. A universal rotavirus vaccination program was implemented and delivered in public health clinics in Prince Edward Island and in physicians’ offices in Nova Scotia.

**Results:**

Engagement of vaccinators in delivery of the universal vaccination program was more successful in Prince Edward Island than in Nova Scotia. Vaccine coverage rates rose rapidly in Prince Edward Island, exceeding 90% for both doses within 3 months and remaining at those levels over the two-year program. In contrast, coverage rates in Nova Scotia rose more slowly and never exceeded 40% during the two years. Access to coverage data was more timely and accurate in Prince Edward Island than Nova Scotia.

**Conclusion:**

A universal rotavirus vaccination program delivered through public health clinics achieved more rapid and higher levels of coverage than a program administered through physicians’ offices.

**Trial registration:**

NCT01273077.

## Background

Rotavirus is the most common cause of infectious gastroenteritis in infants, estimated to cause millions of hospitalizations and from 450,000 to 550,000 deaths annually worldwide [1–3]. Rotavirus infections tend to cause more severe illness in children than other gastrointestinal pathogens; dehydration requiring medical intervention is common [4]. In Canada and other industrialized countries, deaths resulting from rotavirus occur rarely, although hospitalizations, emergency room visits, and physician visits frequently occur [5]. Hospital-based surveillance in Canada by the Immunization Monitoring Program, Active (IMPACT) demonstrated that 48.6% of hospitalized children had dehydration and 7% experienced seizures [6]; most children were previously healthy, without underlying medical conditions.

Two orally administered rotavirus vaccines are available in Canada for the prevention of rotavirus infection: RotaTeq^®^ (Merck Frosst Canada) and Rotarix^®^ (GlaxoSmithKline). Approved in 2006, RotaTeq^®^ is a bovine human reassortant vaccine comprising the P1A [8] genotype and serotypes G1, G2, G3, and G4 [7] administered as a 3-dose schedule. Approved in 2008, Rotarix^®^ is a live, attenuated human G1P1A [8] rotavirus strain administered as a two-dose schedule [8]. Both vaccines were safe and effective in large, field efficacy studies [9, 10]. Both vaccines are available in Canada and are recommended for use by the National Advisory Committee on Immunization (NACI) [11] and for inclusion in universal immunization programs by the Canadian Immunization Committee [12].

Public funding for universal programs did not immediately follow the NACI recommendations because of remaining unknowns about the burden of illness of rotavirus in Canada, the effectiveness of the vaccine in a Canadian context, and the acceptability of a universal program amongst health care providers and the public, despite Canadian data suggesting that the vaccines would be cost-effective [13, 14]. We undertook a population-based intervention in three Canadian jurisdictions to provide additional Canadian data on the feasibility, acceptability, and effectiveness of implementing a publicly funded universal rotavirus vaccination program (URVP). Two implementation methods were compared with a control (no URVP). The objective was to evaluate the logistics of implementing a URVP and compare the effectiveness of the program implementation in jurisdictions with either a physician-administered or public health nurse-administered universal program.

## Methods

### Setting

The study took place in three health regions in the Maritime Provinces. There were two intervention areas (Prince Edward Island [PEI] and Capital District Health Authority of Nova Scotia [CDHA/NS]) and one non-intervention control area (the Saint John zone of the Horizon Health region of New Brunswick [SJ/NB]). In 2011, PEI had an annual birth cohort of 1432 [15] with all publicly funded immunizations provided by public health nurses in public health immunization clinics. The 2011 annual birth cohort of CDHA/NS was estimated to be 4209 (Atlee Perinatal Database, IWK Health Centre, Halifax, NS, personal communication) and most immunizations are provided by family physicians. The birth cohort of SJ/NB was 1757 [16] and most immunizations are provided by family physicians. The study was approved by the Research Ethics Boards of the IWK Health Centre, Halifax, NS, Health PEI, Charlottetown, PEI, and Horizon Health, Saint John, NB. (clinicaltrials.gov NCT01273077).

### Participants and interventions

All infants who resided in PEI and in CDHA/NS born between October 1, 2010 and September 30, 2012 were eligible to participate in the URVP. The non-intervention region, SJ/NB, was included in the surveillance of rotavirus-related hospitalizations but was not considered further in this program implementation analysis. Rotavirus vaccine (Rotarix^®^, GlaxoSmithKline, Canada) was provided to the jurisdictions at no charge. The URVP was implemented according to the standard practices for introducing a new, publicly funded vaccination program. In PEI, this begins with development of a proposal by the Chief Public Health Office Immunization Committee using a decision-making framework that includes multiple programmatic factors including epidemiology and disease burden, nature of the vaccine, and cost-effectiveness [17]. Following consultation with Health PEI regarding implementation issues, the proposed new immunization program typically is submitted to the provincial Treasury Board for funding approval. Once approved, the immunization program is implemented through Public Health Nursing. The implementation process includes an education in-service with immunizing public health nurses, notification of physicians and nurse practitioners of the new program, development and distribution of fact sheets to the public and promotional material to physicians and nurse practitioners, and engagement of the media. In Nova Scotia, vaccine programs are introduced following a recommendation from the Immunization Sub-Committee and approval of the full Communicable Disease Prevention and Control Committee. Proposals for programs are then submitted to the Provincial Treasury Board during the annual budgetary approval cycle. Once government approval is received, information and educational material is distributed to health care providers who administer vaccine (mostly primary care physicians). For the URVP in CDHA/NS, the program decision was made at the district level with provincial support for participating in the demonstration project. Provider education was provided subsequent to an education needs assessment, face-to-face and webinar delivered continuing education sessions, direct interaction and discussion with group practices, engaging physicians in project working groups, mailed and electronic communication with physicians, information sheets included with delivery of other routine pediatric vaccines, and media releases directed at the public and providers. In PEI, rotavirus vaccine was primarily administered along with other routine pediatric vaccines by public health nurses in public health clinics distributed across the province. In CDHA/NS, rotavirus vaccine was provided to family physicians by public health along with other routine pediatric vaccines and was administered in family physicians’ offices. As the rotavirus program was implemented as a public health provided program, consent for vaccination in both jurisdictions was obtained from parents/guardians as per other routinely administered childhood vaccines.

### Outcomes and data analysis

Program effectiveness was monitored by measuring vaccine coverage for the first and second dose of rotavirus vaccine in PEI and CDHA/NS; vaccine coverage was not assessed in SJ/NB where no intervention was undertaken. Coverage was determined by the proportion of eligible infants who received the first and second doses of rotavirus vaccine. In PEI, this was determined using the provincial vaccine registry of doses administered in public health clinics and the annual birth cohort each year. In CDHA/NS, vaccine coverage was measured by the annual birth cohort and the number of doses administered by family physicians as reported either on paper-based or electronic reciprocal notification forms whereby physicians reported who received the vaccine doses provided to them by public health. Insights into the program implementation process were obtained by key informant interviews with public health individuals in PEI and CDHA/NS responsible for implementation of the URVP following completion of the intervention project.

Vaccine impact was evaluated by surveillance for rotavirus-related emergency room visits and hospitalizations in all three jurisdictions but is not included in this report.

## Results

### Pre-implementation activities

In PEI, the program implementation activities were qualitatively similar to other new provincial vaccination programs. The in-service education session for all immunizing public health nurses was attended by 43 (97.7%) of the 44 eligible practitioners. Promotional material about the program was also provided to all physicians and nurse practitioners, although they were not involved in the administration of the vaccine. Fact sheets were provided to parents of all newborn infants and again at public health vaccination clinics. The program was announced in the legislature, through the public health website, and through media releases. In CDHA/NS, two members of the District Department of Family Medicine participated in the program implementation planning committee. Notices describing the new program were sent to all physicians and nurse practitioners and fact sheets were provided at the time of delivery to mothers of all newborn infants. Posters were distributed to physicians’ offices, the public health website was updated, and promotional information was included with all other routine pediatric vaccine orders shipped to physician offices. The needs assessment engaging the 405 practicing physicians in CDHA/NS was completed by 6 (1.5%) practitioners. A face-to-face education session was attended by 8 (2.0%) of the 405 family physicians, and an additional 3 (0.7%) physicians participated in an educational webinar (of note, the webinar was also attended by 10 CDHA/NS public health nurses who were interested in the program but were not involved in administering the vaccine to infants).

### Vaccine coverage

**Figure 1:**
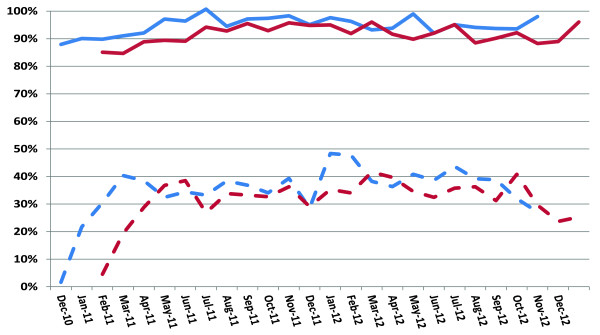
**Vaccine coverage by month for dose 1 (blue lines) and dose 2 (red lines) in PEI (solid lines) and CDHA/NS (dashed lines).**

Vaccine coverage in PEI rose quickly after implementation of the program and was maintained at over 90% during the two-year program (Figure 1). Vaccine coverage for dose one in year one was 94.4% and 91.6% for dose 2. In year two, vaccine coverage for dose one and two was 95.1% and 91.7%, respectively. The slightly lower rates for dose 2 coverage were reported by PEI public health to be due to strict adherence to the age limit for the first and second dose as specified in the vaccine product monograph.In CDHA/NS, according to reciprocal notifications received of doses administered, vaccine coverage rose over the first year to approximately 31.8% for dose one and 29.5% for dose two and continued to increase slowly in year two to 38.1% and 33.7% for doses one and two, respectively (Figure 1). In CDHA/NS, substantially more vaccine was shipped to family physicians’ offices than were reported as administered via reciprocal notification forms, so the vaccine coverage reported is likely an underestimate of the actual vaccine uptake. The maximum vaccine coverage in CDHA/NS by assuming that all shipped doses were administered to eligible infants would be 52.8% in year one and 48.9% in year 2; however, the latter figures are clearly overestimates since they would include vaccine wastage and doses still stored in physicians’ offices.

### Key informant interviews

Telephone interviews were conducted with five key informants from PEI and three from CDHA/NS including four physicians and four nurse public health officials. The implementation of the URVP in PEI was described as smooth and consistent with other public health vaccine programs, rapidly achieving levels of acceptance of established programs. Factors identified as critical to the success were provincial support of the province-wide program, a presumption that the URVP would be continued beyond the term of the project (i.e., introduced as part of the provincially funded routine pediatric vaccination schedule), and public health nurse delivery of the vaccine program through their established, geographically distributed, public health office sites and satellite clinics. The major challenge identified was the novelty of an upper limit to the age of vaccination resulting in some infants being too old for their second dose when they arrived at the office for their 4-month immunizations. This mostly occurred early in the program when staff was adjusting to the strict administration schedule for the vaccine and the time urgency of rescheduling missed appointments. In contrast, program implementation in CDHA/NS was described as challenging, with a number of factors contributing to the low rates of vaccine uptake. Despite substantial efforts to communicate with family physicians using multiple and redundant mechanisms, many physicians continued to be unaware of the program and did not offer vaccine to their patients. While interviewees described this as a common problem with physicians for all new programs, exacerbating factors that were identified included the lack of a province-wide program and that all communication was done by the District (CDHA) without provincial support/reinforcement. Insufficient physician reimbursement for vaccine administration was also identified as a factor resulting in physicians either not offering the vaccine to their patients or providing the vaccine but not reporting back the number of doses administered using reciprocal forms. The lack of presumption that the program would continue after two years was also identified as a potential disincentive for physicians to provide the vaccine to their patients.

## Discussion

This report describes the first URVP implemented in Canada and compares and contrasts the success of two different vaccine delivery systems (public health clinics and family physicians’ offices). In PEI, where rotavirus vaccine was delivered primarily by public health nurses in province-wide public health offices and satellite locations, implementation proceeded smoothly and vaccine uptake was rapid, quickly achieving coverage rates in excess of 90% for both the first and second doses. In contrast, in CDHA/NS where vaccine was administered in family physician offices, program implementation was plagued by lack of awareness by physicians and care-givers, despite extensive efforts to engage their participation, inform them about the program, and provide them with the necessary tools to provide the vaccine to their patients. As a result, vaccine uptake was suboptimal and, at the end of the two-year program, still lagged far below other routine pediatric immunizations. A potential contributing factor to the better vaccine coverage in PEI compared to CDHA/NS is that the program in PEI was province-wide whereas in NS it was limited to a single district. However, CDHA/NS is home to nearly 50% of the provincial population in NS and the implementation methods employed mirrored those methods routinely used for province-wide programs. Although the vaccine was provided to physicians at no cost, there were concerns about inadequate compensation to the physicians for vaccine administration. Because rotavirus vaccine was the first orally delivered vaccine available in NS, there was no established reimbursement rate for this activity. In consultation with local physician leaders, a rate of 50% of the rate provided for injectable vaccines was established; however, this may not have been deemed sufficient by some practicing physicians.

In comparing the implementation successes of the two programs, it may be that, despite the logistical barriers, the vaccine coverage achieved in CDHA/NS may be typical for new vaccine programs in a physician-delivered or mixed public health/physician-delivered system. In the United States, rotavirus was recommended for universal infant programs in 2006 [18]. In 2009, the first year for which data are available in the National Immunization Survey, vaccine coverage was only 43.9% among children 19–36 months of age, rising to 59.2% in 2010, 67.3% in 2011, and 68.6% in 2012 [19]. This slow rate of increase in vaccine coverage is typically observed in the US. Human papillomavirus vaccine (HPV) was recommended for universal immunization of pre-adolescent girls in the US in 2007 [20] and in 2008, vaccine coverage for the first dose was 37.2% and the third dose 17.9%. Coverage rates rose by 5–10% per year and by 2011 coverage was reported as 53.0% for dose 1 and 34.8% for dose 3; no further increase in coverage was reported in 2012 [21]. Similar coverage rates for the first and subsequent years of universal programs have been reported in the US for other vaccines including pneumococcal conjugate and varicella vaccines [22]. In contrast, in Finland, vaccinations are given by public health nurses in public health clinics or in schools [23]. Rotavirus vaccine was offered to all infants beginning in September 2009 and achieved vaccine coverage rates in excess of 95% during the first two years of the program, comparable to other vaccines in their universal infant program [24].

In Nova Scotia where human papillomavirus vaccine is administered by public health nurses in school-based programs, vaccine coverage rates after the first three doses was 85.3%, 81.2%, and 77.1%, respectively in 2008, one year after implementation of the program [25]. Three years later in 2011, coverage rates had risen to 92.3%, 83.4%, and 76.1% for the three doses. No data are available about the coverage rates in Nova Scotia of other recently introduced physician-administered publicly funded vaccine programs (conjugate meningococcal vaccine, varicella vaccine, pneumococcal conjugate vaccine). In PEI, vaccine coverage with the rotavirus vaccine is similar to their success with other recent universal vaccine programs, all of which are administered by public health nurses. For example, with meningococcal conjugate vaccine given to infants at 12 months of age, vaccine coverage was 93% in the year following implementation of the program and has maintained a level of at least 90% since that time. With HPV vaccine, coverage rates after the first three doses was 87.6%, 84.9%, and 81.1%, respectively, one year after implementation of the program. Four years later, coverage rates had risen to 90.7%, 90.3%, and 87.3% for the three doses.

Rapid uptake of vaccines in publicly funded programs can be successfully accomplished in physician office-delivered programs. In the United Kingdom where childhood vaccines are provided by general practitioners, pneumococcal conjugate vaccine was introduced into the universal vaccination program in 2006 and vaccine coverage of 83.7% was achieved for the primary series by 12 months of age after the first year, rising to 91% in the second year [26]. The nature and perception of the infection, disease, and vaccine may play a role in how rapidly vaccine coverage is achieved. Vaccine coverage targets for practices and financial incentives for meeting those targets may also contribute to the success observed in the UK [27]. Rotavirus vaccine was introduced into the UK publicly funded vaccine program in 2013; first-year coverage data won’t be available until late 2014.

Although high rates of vaccine coverage can be achieved in physician-delivered programs, as we found in this study, the rise in vaccine coverage rates appears to be more uniformly rapid in public health nurse-delivered programs. An additional benefit of public health delivered programs is the precision of vaccine coverage estimates, particularly in the absence of vaccine registries. In CDHA/NS, the rotavirus vaccine coverage rates achieved are bracketed between vaccine doses that were reported as given and vaccine doses that were provided to physicians. In contrast, the coverage rates provided by PEI are precisely as reported.

As a result of the high coverage rates achieved in PEI, hospitalizations related to rotavirus virtually disappeared from the province. In contrast, there was a less dramatic effect of the URVP in CDHA/NS (unpublished data, manuscript submitted). This may be a contributing factor to the decision by the PEI Department of Health and Wellness to continue the URVP after completion of this project. In contrast, NS remains one of 5 Canadian provinces without a URVP.

## Conclusion

In Canada, there is no uniformity about how childhood vaccines are delivered among the provinces or even by district within a province. For example, childhood vaccines are delivered primarily by public health nurses in PEI and Alberta whereas in Nova Scotia and New Brunswick, vaccines are provided by public health nurses in some districts and physicians in other districts. Although the programmatic advantages of public health delivered childhood vaccines appears clear, questions about the relative costs of the two delivery systems or the costs of changing from a physician-delivered to a public health nurse-delivered program have not been established. Public attitudes and preferences also have not been fully explored and, given the increasing numbers and complexities of the childhood vaccination schedule, it is difficult to determine what provider preferences are for the ideal vaccine delivery system for the future. Further research and analysis regarding the appropriate use of resources, personnel, cost, outcomes, and accessibility are needed.
